# Effectiveness of a Nutrition Counseling Intervention on Food Consumption, According to the Degree of Processing: A Community-Based Non-Randomized Trial of Quilombola Communities in South Brazil

**DOI:** 10.3389/ijph.2024.1607549

**Published:** 2024-11-27

**Authors:** Pauline Müller Pacheco, Fernanda de Souza Bairros, Marilda Borges Neutzling, Luciana Neves Nunes, Daniela Riva Knauth, Francine Silva dos Santos, Michele Drehmer

**Affiliations:** ^1^ Postgraduate Studies Program in Epidemiology, School of Medicine, Federal University of Rio Grande do Sul, Porto Alegre, Brazil; ^2^ Postgraduate Studies Program in Collective Health, Federal University of Rio Grande do Sul, Porto Alegre, Brazil; ^3^ Department of Statistics, Federal University of Rio Grande do Sul, Porto Alegre, Brazil; ^4^ Department of Nutrition, Federal University of Health Science of Porto Alegre, Porto Alegre, Rio Grande do Sul, Brazil; ^5^ Postgraduate Studies Program in Food, Nutrition and Health, School of Medicine, Federal University of Rio Grande do Sul, Porto Alegre, Brazil

**Keywords:** quilombola communities, nutritional counseling, dietary intake, food processing, NOVA classification

## Abstract

**Objectives:**

To evaluate the effectiveness of a nutrition counseling intervention on food consumption according to the Nova classification that reflects levels of food processing.

**Methods:**

Controlled community trial was conducted in quilombola communities in the South of Brazil. Four communities were allocated to the control group (CG) and the intervention group (IG), two communities comprised each group. A total of 158 individuals (CG = 87; IG = 68) were included in the study. The intervention consisted of six theoretical and practical workshops on food and nutrition education, conducted over a 4-month period. We used a 24-hour recall at baseline and another post-intervention.

**Results:**

There was an increase in the consumption of traditional quilombola food as an effect of the intervention (from 14.5% to 20.7% in the IG, and from 12.7% to 16.0% in the CG, *p* = 0.05). There was no significant variation in the other Nova food groups according to time and intervention.

**Conclusion:**

An increase in traditional quilombola food indicates a resumption of traditional food intake and appreciation of local culture as an effect of this intervention at the community level.

**Clinical Trial Registration:** www.clinicaltrials.gov, identifier NCT02489149.

## Introduction

The Nova system proposes the classification of foods based on the extent and purpose of the industrial processing [1]. Since its first publication, more than a decade ago [2], the Nova system has contributed to nutritional epidemiology and public health, by including other food dimensions into the dominant nutrient-based approach, such as ingredients and processing characteristics [3, 4]. At the level of foods, has been considered ingredients, processing characteristics, and impact on dietary patterns [4]. Although the Nova system classifies the foods into four food groups, the ultra-processed (Nova group 4) has received the main focus in the literature. These foods are industrial formulations with ingredients that might not be available for domestic use, ready for consumption, elaborated - entirely or mostly - from substances extracted from food or synthesized in the laboratory, with little or no intact food [5]. These products are normally nutritionally unbalanced and offer advantages for their convenience, durability, and hyper palatability. In addition, they have great sales profit, due to the low cost of ingredients, made in conjunction with attractive packaging and aggressive advertising [1, 5].

The trend toward increasing ultra-processed foods consumption is accompanied by the replacement of the traditional diet [1, 6]. In other words, the Nova group 1 (unprocessed and minimally processed foods) and Nova group 3 (processed foods) have been replaced by ultra-processed foods [4]. According to population-based data, the Nova group 1 still represents the base of the Brazilian diet [7]. However, the participation of ultra-processed foods in the total calories determined by household food purchases increased from 14.3 in 2002/03 to 17.3% and 19.4% between 2008/09 and 2017/18, respectively [7]. Furthermore, a growing body of evidence links ultra-processed foods to an increased risk of obesity, chronic disease, and mortality [8–10]. It has been recently estimated that more than a fourth of the increase in the prevalence of obesity in Brazil between 2002 and 2009 was attributable to ultra-processed foods [11].

In this context, the Dietary Guideline for the Brazilian Population, the main instrument to promote healthy eating practices in the country and updated in 2014, incorporated the Nova system as the guide for food choices, bringing as the golden rule always prefer natural or minimally processed foods and freshly made dishes and meals to ultra-processed food [12]. Also, the document points out the existence of population groups more vulnerable to nutritional issues, such as the quilombolas, and emphasizes the dialogue with sociocultural, economic, and environmentally sustainable dimensions [12].

The quilombola communities are racial/ethnic groups of black ancestry, originating during the period of slavery and after abolition. They usually, but not exclusively, live in isolated territories as a form of resistance to racial/ethnic oppression [13]. In the quilombola communities of the State of Rio Grande do Sul in the South of Brazil, homemade foods play a key role, including bakery products and traditional meals based on rice and beans [14]. However, the replacement of traditional foods by ultra-processed in quilombola communities has been observed [15].

Concerning food consumption based on the Nova system, there is a gap in national population-based data from quilombola communities. A study conducted in 14 Brazilian States showed that sweets (including candies, gelatin, and ice cream), packaged snacks, canned or inlaid meat, chocolate milk, ready-made cookies and cakes, and soft drinks or powdered soft drinks were purchased in the previous week by almost all families, including those in extreme poverty [16]. In the state of Rio Grande do Sul, the same locality where this study was conducted, ultra-processed represented the main options for snacks, including cookies (31.4%), packaged salty snacks (10.2%), and candies (9.3%) [17]. Thus, these data suggest a nutritional transition framework for quilombolas’ diet [15].

A nutrition counseling nutrition interventions in quilombola communities should focus on the promotion of traditional food habits, since cooking activities is a mechanism of cultural resistance, and actions to mitigate ultra-processed foods consumption [15]. As historically, quilombola communities are a vulnerable group to food and nutrition insecurity, the growing share of ultra-processed foods in the diet of these communities, particularly due to the above-mentioned characteristics of the ultra-processed foods, can lead to the double burden of malnutrition [4]. Therefore, the current study aimed to evaluate the effectiveness of a nutritional counseling intervention on food consumption, according to the Nova system food classification in quilombola communities in the South of Brazil.

## Methods

### Design and Sampling

The current study is a non-randomized, controlled, parallel, cluster-type intervention conducted in four quilombola communities in the South of Brazil. Thus, it is in accordance with the TREND statement’s recommendations for behavioral and public health interventions involving non-randomized research [18].

The studied sample originated from the main research project entitled “Food and Nutrition Education in quilombola communities with food insecurity: rescue of food culture, promotion of healthy eating and the demand for the Human Right to Food.” It was carried out from 2014 to 2016. The four quilombola communities of the Rio Grande do Sul that had the highest prevalence of overweight and food insecurity, according to a population-based study [19] and, considering the logistic and financial capacity of the study, were eligible to participate in his project. Two communities were assigned to the intervention group (IG) and control group (CG), respectively. Supplementary Figure S1 shows the location of these quilombola communities in the same region of the State of Rio Grande do Sul (Canguçu, Pelotas, Cristal, and Nova Palma).

These cities are located in the extreme south of Brazil, with an agricultural economic tradition, and formed from large farms that used the labor of individuals enslaved in the 18th and 19th centuries. Besides, the communities included in this study are located in the rural areas of the cities mentioned above. They have low coverage of health services, low education levels [14], important illiteracy and unemployment rates, precarious housing conditions, and a high prevalence of chronic non-communicable diseases such as hypertension [20], diabetes, and overweight [14].

All self-declared heads of households in these four communities, located in the rural areas of the municipalities of Pelotas (n = 61), Canguçu (n = 35), Cristal (n = 42), and Nova Palma (n = 40), totaling 178 individuals, were included in the study. Of these, two communities received an educational nutritional intervention (Passo do Lourenço and Algodão communities, in the municipalities of Canguçu and Pelotas, respectively), and two did not, serving as control groups (Serrinha do Cristal and Rincão do Santo Inácio, in the municipalities of Cristal and Nova Palma, respectively).

### Study Design

**FIGURE 1:**
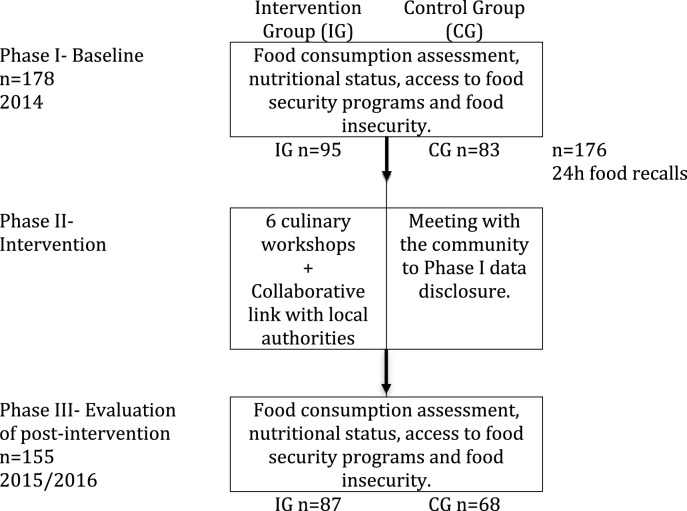
Flowchart of the study design conducted in four quilombola communities in Rio Grande do Sul, Brazil, 2014–2016.

This study was registered at ClinicalTrials.gov under the identifier: NCT02489149. Figure 1 presents the study phases. Phase I refers to the baseline, the intervention was performed in phase II, and phase III is the post-intervention evaluation.

In Phase I, quantitative and qualitative diagnosis was performed. The quantitative diagnosis consisted of a census-type survey involving all those responsible for families according to self-attribution criteria of the intervention communities and controls on their food consumption, nutritional status, access to programs to fight hunger, and the prevalence of food insecurity, measured by Ebia scale [21]. For qualitative data collection, focus groups were conducted to assess traditional habits and recipes.

Intervention strategies for Phase II were developed based on Phase I results and were conducted over a 4-month period. Workshops were carried out with the community members to form multipliers (Supplementary Table S1). The workshops are detailed in their methodology and materials for their reproduction in a booklet that was produced and then distributed to all participating families [22]. Actions with the institutional public, including municipal managers - from the areas of health, education, culture and rural development, as well as health professionals from the Family Health Strategy teams in the region, were carried out to minimize food insecurity and promote healthy eating.

### Instruments and Data Collection

For phases I and III, standard pre-coded and pre-tested questionnaires were employed. Questions about socioeconomic and demographic features including age, education, sex, skin color, income, physical activity, access to health services, social development, and benefit from social programs were answered by the person responsible for the self-assigned household at the time of the interview. Trained interviewers measured the nutritional status of household heads. The scale used was from the Marte^®^ brand with a capacity of 200 kg and an accuracy of 50g, along with Exact Height^®^ brand anthropometers with an accuracy of 1 mm. The BMI was calculated as the weight in kilograms divided by the height in squared meters (kg/m^2^) [23]. The interviewers underwent extensive training to administer the questionnaire and perform anthropometric measurements. A manual was created and provided for quick reference, containing descriptions of all variables, alternative questions for instances where interviewees did not understand the initial question, and guidance on coding responses. Physical activity was evaluated by International Physical Activity Questionnaire (IPAQ) short form that asks about three specific types of activity undertaken in the three domains: walking, moderate-intensity activities and vigorous intensity activities; frequency (measured in days per week) and duration (time per day) are collected separately for each specific type of activity. A combined total physical activity metabolic equivalent-minute per week (MET-min/week) was computed as the sum of walking + moderate + vigorous MET-min/week scores [24].

To assess dietary intake, trained and standardized interviewers applied two 24-hour recalls: one at baseline and another post-intervention. In addition to the quantities (in homemade measures), the 24-hour recall included information on the food brands, the form of preparation, the place where the food was obtained (inside or outside the home), the time of consumption, whether the food was classified as diet or light (any food or beverage whose recipe is altered to reduce fat, carbohydrates, and sugar for example), and whether it was classified as homemade, ready for consumption or ready to heat, facilitating the subsequent classification according to food processing.

The Automated Multiple-Pass Method [25] was employed to obtain more accurate information concerning food consumption. This method comprises five steps designed to minimize memory bias in 24-hour recall reporting. Additional questions were asked using a conference list of commonly forgotten products and, finally, the recipes for the preparations were detailed. A photographic album of homemade measurements was used to estimate the quantities [26]. In addition, the interview was conducted preferably in the interviewee’s home and kitchen, where the utensils, and the brand of food consumed could be observed. The questionnaires, after being reviewed and coded by the field supervisor, were entered with double check of the data.

The data obtained in the study were double-entered in the Epidata software version 3.1 and the 24-hour dietary recalls in ADS Nutri [27]. The Brazilian Table of Food Composition – TACO was used as a reference for energy and nutrient calculations.

### Outcome

The outcome measured was the percentage of energy contribution from the different Nova system groups. This system classifies food into four categories based on the extent and purpose of the industrial processing: unprocessed or minimally processed foods (Group 1), processed culinary ingredients (Group 2), processed foods (Group 3), and ultra-processed foods (Group 4) [1]. Our analysis adheres to the original four groups outlined by the Nova classification. However, due to the cultural traditions of the quilombola population, the results also distinguish “traditional quilombola food,” which is separated from the processed foods group.

Traditional quilombola food (included in Nova Group 3) refers to preparations made at home using unprocessed or minimally processed foods (Group 1) and culinary ingredients (Group 2). Examples include homemade bread, cakes, bread rolls, and recipes incorporating vegetables such as carrots, pumpkin, and corn, as well as dishes like beans with canjica, cornmeal with meat, guacamole, and oven-baked sweet potatoes, among others [12].

### Data Analysis

Descriptive data analysis was performed to characterize and compare the CG and IG at baseline through frequency distribution, means and standard deviation (SD). Pearson’s chi-square and Student’s T-test were used to test differences between the categorical and continuous variables, respectively. The outcome was used as a continuous variable. For comparison of the energy contribution from the Nova food groups between groups, before and after intervention, the Generalized Estimation Equations model was used. We estimated the effect of belonging to a group (intervention or control), the time in between the first and third phases, and the interaction of these two factors.

The robust estimator was employed with an unstructured work matrix and normal distribution with identity binding function. The *post hoc* test adopted was the Bonferroni test. The absence of data in the third phase of the study was treated as loss (missing) without data imputation. In all comparisons, a *p*-value <0.05 was considered significant. Data analysis was performed using the IBM SPSS statistical software, version 18.0.

### Ethical Aspects

The study was approved by the Human Research Ethics Committee of the Federal University of Rio Grande do Sul (UFRGS) and all participants signed the Informed Consent Form. In addition to the measures mentioned, as this is a study in traditional communities, previous contacts were also made with the leaders of the communities, to expose the research goals and obtain their consent.

## Results

All those responsible for the families of four communities, two communities that participated in the intervention actions and two control communities were evaluated, totaling 178 adults interviewed and 176 24-hour food recalls collected. In the final evaluation (phase III), 21 individuals (11.8%) were lost to follow-up. Thus, the final sample comprised 158 individuals (CG = 87, IG = 68).

Table 1 shows the sociodemographic, anthropometric and food and nutritional security characteristics of those responsible for the families at the baseline, as well as, the differences between the IG and CG. A difference was found in the food and nutritional security variable. In the IG communities the moderate and severe food insecurity was greater than in CG communities of the IG (58.9% vs. 25.3%).

**TABLE 1:** Demographic, socioeconomic, anthropometric characteristics, food and nutritional security of those responsible for the families of quilombola communities in Rio Grande do Sul at the baseline, Rio Grande do Sul, Brazil, 2014 (n = 178).

Variables	Intervention group (n = 95)	Control group (n = 83)	*P*-value
Sex (female)	66.3%	73.5%	0.30
Age (years)	45.4 ± 15.6	44.1 ± 16.5	0.60
Race/color (black or brown)	91.6%	85.5%	0.20
Schooling (years)	4.2 ± 5.4	4.6 ± 4.1	0.64
Family monthly income (reais)	819.0 ± 954.8	903.2 ± 638.8	0.50
Per capita monthly income (reais)	280.3 ± 2,990	317.7 ± 269.7	0.38
Has a signed work card	2.7%	1.7%	0.71
Inhabitants per house hold	3.73 ± 2.26	3.61 ± 1.75	0.72
BMI (kg/m^2^)	28.65 ± 5.97	28.07 ± 5.65	0.53
Physical activity (MET-min/week)	7,620 (3,066–15,360)	7,332 (2,739–15,090)	0.76
Food and nutritional security			<0.01
Security/Low insecurity	41.1%	74.7%	
Moderate insecurity/Severe	58.9%	25.3%	
Access to cash transfer program	57.9%	47.0%	0.15
Consumption of food produced by the family	55.8%	68.7%	0.09

*Results expressed by mean ± SD, frequency (%), and median (P25 - P75). Student T-test; chi-square or Mann-Whitney *U* Test; *p* < 0.05.

Abbreviations: BMI, body mass index.

Physical activity: combined total physical activity MET-min/week computed as the sum of Walking + Moderate + Vigorous MET-min/week scores.

Table 2 shows the baseline dietary intake according to allocation group. There was no significant difference in the mean daily total energy (1,821.7 ± 955.8 kcal/d in the IG, 1,825.1 ± 824.9 kcal/d in the CG) and proteins, fibers, and sodium. The percentage of contribution to the total calories from unprocessed or minimally processed foods was approximately 52% for both groups, processed culinary ingredients (16.1% in the IG, 19.8% in the CG), processed foods (16.8% in the IG, 14.4% in the CG), and ultra-processed foods (13.9% in the IG, 15.0% in the CG).

**TABLE 2:** Contribution in grams and percentage of total calories per day from Nova food groups, traditional quilombola food, and nutrient consumption by those responsible for the quilombola communities in Rio Grande do Sul on the baseline study, Rio Grande do Sul, Brazil, 2014 (n = 176).

	Intervention group (n = 95) Mean ± SD		Control group (n = 83) Mean ± SD		*P*-value
	Kcal/d or g/day	%TEV/day	Kcal/d or g/day	%TEV/day	
Unprocessed or minimally processed foods^a^	1383.7 ± 953.7	52.3 ± 20.7	1289.0 ± 775.3	52.0 ± 21.4	0.92^a^
Processed culinary ingredients^a^	184.9 ± 175.9	16.1 ± 13.4	160.6 ± 157.4	19.8 ± 14.3	0.07^a^
Processed foods^a^ ^,^ ^b^	141.6 ± 153.6	16.8 ± 13.6	113.4 ± 117.8	14.4 ± 11.8	0.64^a^
Traditional quilombola food^a^	102.6 ± 103.4	14.5 ± 12.4	85.8 ± 85.4	12.7 ± 11.3	0.32^a^
Ultra-processed foods^a^	106.2 ± 164.7	13.9 ± 15.4	147.3 ± 219.1	15.0 ± 19.2	0.68^a^
Energy (Kcal/d)	1821.7 ± 955.8	—	1825.1 ± 824.9	—	0.98
Protein (g/d)	71.5 ± 44.3	15.8 ± 6.4	72.5 ± 48.9	16.0 ± 7.3	0.89
Carbohydrate (g/d)	213.7 ± 110.5	49.8 ± 15.9	221.2 ± 111.3	49.7 ± 15.8	0.91
Fat (g/d)	71.9 ± 61.3	33.0 ± 13.5	70.6 ± 47.6	33.0 ± 13.5	0.65
Fiber (g/d)	22.2 ± 13.7	—	21.2 ± 13.8	—	0.63
Sodium (mg/d)	3634.3 ± 4838.4	—	3336.8 ± 2883.7	—	0.63

%TEV: percentage of the total energy value.

^a^ P value for the difference of the means of the percentage of the total energy consumption.

^b^ Including culinary preparations according to original Nova classification.

Table 3 shows the percentage values of energy contribution of the Nova food groups before and after the intervention, according to allocation group. Any food group presented statistically significant variation as a function of the effect of the intervention, except traditional quilombola food that presented an increase in consumption from 14.5% to 20.7% in the IG, and from 12.7% to 16.0% in the CG (*p* = 0.05) as an effect of the intervention. There was a variation in the percentage of the energy contribution from the several food groups, in the CG and IG, attributed to a variation in time regardless of the intervention. The group of unprocessed or minimally processed foods (from 52.3% to 45.6% in the IG, and from 52.0% to 49.0% in the CG, *p* = 0.01) and culinary ingredients (from 17.8% to 13.2% in the IG, and from 18.1% to 13.8% in the CG, *p* < 0.01) had a reduction in the percentage of contribution as an effect of time. In contrast, processed foods, traditional quilombola food, and ultra-processed foods increased (significance values for the effect of time related to the data in Table 3).

**TABLE 3:** Changes in the percentage of the total energy value of consumption of unprocessed or minimally processed foods, culinary ingredients, processed foods, ultra-processed foods and traditional quilombola food between the pre and post intervention periods of those responsible for the family of Quilombola communities in Rio Grande do Sul, Rio Grande do Sul, Brazil, 2014 and 2016 (n = 155).

	Phase 1		Phase 3		*P*-value for intervention	*P*-value for time	*P*-value for interaction (intervention*time)
	Intervention (n = 93)	Controls (n = 83)	Intervention (n = 87)	Controls (n = 68)			
	%TEV		%TEV				
Unprocessed or minimally processed foods	52.3 ± 20.7	52.0 ± 21.4	45.6 ± 16.5	49.0 ± 21.7	0.54	0.01	0.35
Culinary ingredients	17.8 ± 14.7	18.1 ± 13.0	13.2 ± 8.8	13.8 ± 10.2	0.73	<0.01	0.87
Processed foods^a^	16.8 ± 13.6	14.4 ± 11.8	22.3 ± 18.2	19.7 ± 16.5	0.24	<0.01	0.74
Traditional quilombola food	14.5 ± 12.4	12.7 ± 11.3	20.7 ± 18.6	16.0 ± 15.7	0.05	<0.01	0.34
Ultra-processed foods	13.9 ± 15.4	15.0 ± 19.2	18.6 ± 15.5	17.7 ± 20.1	0.97	0.02	0.55

%TEV: Percentage of the total energy value.

Generalized Estimation Equation Model; Bonferroni.

Values expressed by means ±SD.

*P* values expressed for the effects of the intervention, time and interaction between intervention and time.

^a^ Including culinary preparations according to original Nova classification.

Table 4 shows total energy among the groups and subgroups of the Nova classification. We found a reduction in the contribution of total calories per day, as well as calories consumed through meat and fruit. In the evaluation of subgroups, a significant difference was found between the means of consumption from phase I to phase III for meat, rice, beans, roots and tubers, oil and sweetened beverages that include soft drinks. The percentage contribution from meat, oil and roots and tubers was reduced. Meat varied due to time, as did rice presenting a reduction and beans presenting an increase. Roots and tubers, vegetable oils were reduced according to time and intervention. In contrast, the intake of traditional quilombola foods and soft drinks increased with both time and intervention.

**TABLE 4:** Distribution of total energy among the groups and subgroups of the Nova classification by those responsible for the family of quilombola communities in Rio Grande do Sul of the Intervention group, Rio Grande do Sul, Brazil, 2014 (n = 155).

Food subgroup	Phase 1		Phase 3	
	kcal/d Mean ± SD	% of TEV Mean ± SD	kcal/d Mean ± SD	% of TEV Mean ± SD
Total energy value	1821.7 ± 955.8	—	1538.8 ± 758.4	—
Group1: fresh or minimally processed foods	952.7 ± 197.8	52.3 ± 20.7	800.2 ± 162.3	52.0 ± 21.4
Beef, poultry, fish and seafood, eggs	385.2 ± 399.8	19.6 ± 16.8	229.8 ± 238.2^i^	14.1 ± 11.6
Rice and other cereals (including pasta and other pasta)	216.8 ± 189.2	12.7 ± 9.0	212.6 ± 183.2^i^	14.2 ± 10.7
Beans and other legumes	94.8 ± 73.9	6.7 ± 6.5	105.2 ± 91.0^i^	7.7 ± 7.2
Roots and Tubers	50.8 ± 95.4	3.2 ± 6.6	20.0 ± 54.8^j^	1.3 ± 3.6
Milk and natural yogurt	32.9 ± 69.0	1.9 ± 4.3	29.4 ± 57.7	1.9 ± 4.0
Fruits and fruit juices	117.7 ± 200.8	6.5 ± 10.2	75.1 ± 129.4	5.3 ± 8.3
Legumes	17.3 ± 27.1	1.2 ± 2.4	15.5 ± 23.6	1.0 ± 1.4
Others^a^	9.1 ± 34.6	0.6 ± 2.0	3.6 ± 4.9	0.3 ± 0.5
Group 2: processed culinary ingredients	293.3 ± 128.1	16.1 ± 13.4	304.7 ± 108.4	19.8 ± 14.3
Table sugar^b^	90.8 ± 135.0	4.9 ± 7.4	86.7 ± 130.8	5.4 ± 6.8
Vegetable oils	162.4 ± 317.9	7.7 ± 9.8	74.1 ± 64.8^j^	5.7 ± 5.2
Other culinary ingredients^c^	52.1 ± 82.3	3.5 ± 6.6	43.3 ± 62.0	2.7 ± 3.8
Group 3: processed foods	306.0 ± 129.9	16.8 ± 13.6	221.6 ± 89.5	14.4 ± 11.8
Traditional quilombola food	264.0 ± 304.1	14.5 ± 12.4	314.3 ± 309.2^j^	20.5 ± 18.7
Fresh bread	1.7 ± 16.1	0.2 ± 1.6	0	0
Ham and other salty meats, smoked or canned	0	0	0	0
Cheese	0	0	1.3 ± 8.7	0.1 ± 0.5
Canned vegetables	0.7 ± 4.1	0.02 ± 0.1	0.7 ± 5.0	0.1 ± 1.0
Others^d^	42.9 ± 112.8	2.1 ± 4.9	18.0 ± 63.8	1.0 ± 2.9
Group 4: ultra-processed foods	253.2 ± 147.2	13.9 ± 15.4	238.5 ± 145.6	15.0 ± 19.2
Candies^e^	36.2 ± 83.0	1.9 ± 4.2	64.0 ± 175.9	3.2 ± 7.5
Pizzas, hamburgers and sandwiches	17.0 ± 110.5	0.7 ± 4.4	6.4 ± 30.4	0.6 ± 3.3
Sweetened drinks	20.7 ± 50.4	1.1 ± 2.6	35.5 ± 67.3^j^	2.1 ± 4.5
Salty snacks^f^	27.5 ± 103.5	1.3 ± 4.2	26.6 ± 110.1	1.7 ± 6.7
Frozen dishes, “instant” and long-lasting^g^	0.7 ± 6.7	0.1 ± 0.8	8.1 ± 46.6	1.0 ± 5.3
Reconstituted meat and fish products	54.6 ± 170.1	3.1 ± 9.9	57.8 ± 131.6	3.6 ± 7.1
Ultra-processed breads and breakfast cereals	30.0 ± 94.9	2.3 ± 8.3	29.0 ± 87.6	2.1 ± 6.4
Others^h^	67.4 ± 127.7	3.5 ± 6.7	81.5 ± 141.3	4.5 ± 6.2

%TEV: percentage of the total energy value.

^a^ Oil seeds, coffee, tea and ferment.

^b^ Including honey.

^c^ Including animal fats like butter, lard, sour cream, and vinegar.

^d^ Salted or caramelized oilseeds, beer, and wine.

^e^ Cookies, cakes, sweet bakery products, candies, lollipops, chocolate, gelatin, ice cream and other industrialized desserts.

^f^ Including crackers and processed snacks.

^g^ Including instant or canned soups, or ready-made pasta dishes.

^h^ Margarine, ready-made sauces, soy-based products and distilled alcoholic beverages.

^i^ Analysis of the Generalized Estimation Equation Model showed a statistically significant difference attributed to time.

^j^ Analysis of the Generalized Estimation Equation Model showed a statistically significant difference attributed to time and the intervention.

## Discussion

This community-based non-randomized trial evaluated the effectiveness of a nutritional counseling intervention on food consumption, according to the degree of processing in quilombola communities. It was noted that the consumption of traditional quilombola food (part of the Nova group 3) potentially increased after intervention, suggesting that actions at the community level may promote healthy eating and rescue the traditional food culture.

In the workshops carried out during the intervention, traditional recipes identified during the research were prepared in the focus groups, encouraging the use of traditional ingredients such as sweet potatoes, canjica, beans, and pork. Previous studies found that community interventions focused on the development of culinary skills [28–30] obtained significant results in the increase in the frequency and daily amount of vegetable consumption [29, 30]. Also, the development or perpetuation of culinary skills – necessary for the preparation of traditional dishes – are positively associated with a better quality of diet and inversely related to the consumption of ultra-processed [31].

Other experiences with community interventions of quasi-experimental methodology are found [32, 33] aimed at traditional populations [32, 34–36] or with an approach to the cultural perspective of the communities [37]. Browne et al. [32] explored eleven systematic reviews on nutritional education interventions in Australian Aboriginal communities. The findings suggest the success of the interventions occurred mainly when there was community involvement in the development and implementation of programs, including programs with complex approaches that integrate knowledge, skills, and access to healthy foods, nutritional education with a community approach, and workshops on culinary skills [32].

Similarly, interventions aimed at black communities [37], native populations of North America [34], Alaska [35], and Ecuadorian indigenous people [36] argue in favor of incorporating cultural aspects of the community, with actions developed in collaboration with the target sample, as a relevant aspect for the success of the initiatives, as well as for the sustainability of the results of promoting healthy eating over time. Collectively oriented interventions, with the potential to stimulate a healthier environment and circumvent voluntary behavior changes, are capable of causing changes in food consumption in order to reduce inequities [38]. On the other hand, nutritional interventions focused on individual orientations can be exclusionary and especially affect those with greater socioeconomic disadvantages [38, 39].

We did not find significant changes in the other Nova food groups. In the present study, the consumption of ultra-processed foods was lower than that reported for the general Brazilian population. According to data from the National Food Survey (INA), based on the personal food consumption module applied to a subsample of households from the 2017–2018 Household Budget Survey (POF), the average daily *per capita* energy contribution from ultra-processed foods was 19.7% [40]. Additionally, between 2008–2009 and 2017–2018, this percentage increased by 2.04 percentage points among Black individuals, but not among White individuals [41]. At baseline in the present study, ultra-processed food consumption contributed 15% or less to the total energy intake in both the intervention and control groups. Although the POF results are not representative of traditional communities, such as quilombolas, the trends observed in the Black population highlight an increasing consumption of ultra-processed products, driven by a complex food system. This growing trend presents challenges for interventions aimed at reducing the consumption of ultra-processed foods in vulnerable populations, particularly when considering the low daily fiber intake and sodium levels exceeding recommended guidelines for adults in these groups [42, 43].

Furthermore, there was a reduced total energy daily from baseline to post-intervention period, which may affect the distribution of the percentual of energy from the Nova food groups. The reduction in the average consumption of total energy from unprocessed and minimally processed foods may be caused by a significant reduction in the energy obtained from meat and eggs. Among the Nova group 1, the mentioned foods may provide the greatest energy density, as well as, they have a high monetary cost [44]. In contrast, ultra-processed foods usually present high energy density [5] contributing to the growing participation of these products in the total daily energy in consequence of reducing foods of Nova group 1.

In this sense, an important barrier to established changes in food consumption among quilombola communities might be the economic dimension. Traditional communities, such as the quilombolas, have become a target of the big food industry since they represent the social stratus with the lowest participation of these products in the diet. It may indicate an emergent framework of inequalities in food consumption. Since the traditional communities have been exposed to food with low nutritional quality which may lead to diseases related to nutritional deficiencies, as well as, overweight/obesity and other chronic diseases [4]. Also, predictions of the food prices in Brazil suggest that ultra-processed foods can be cheaper than unprocessed and minimally processed in the next years [45]. Among the Nova group 1, the “staple foods” as rice, been, and cassava are the main representants of this group in the traditional diet of the quilombolas [15–17], the reason could be because they are more economically available than other fresh foods (i.e., fruits and vegetables).

Another construct that could be a barrier to healthy changes in the quilombola diet is the food environment [46], as the low availability of healthy foods. We observed in these communities that there is low availability of vegetables for purchase, while variety and quantity are abundant of ultra-processed, such as soft drinks and snacks. As the quilombola communities included in this study are located in areas far from urban centers, they comprise a typical food desert, usually found in low-income urban or rural areas where the closest source of nutritious food is far away or difficult to access due to a lack of transportation, where residents have limited access to affordable, nutritious food, particularly fresh fruits, vegetables, and other whole foods necessary for a healthy diet, [46, 47]. Thus, even if individuals have the resources to buy fruits and vegetables, they do not find quality products, and at the same time, they are exposed to several advertising stimuli to ultra-processed.

Strengths of this study relate to its design, the sample of a traditional community with scarce data in the literature, and the intervention was developed in collaboration with several actors (people from the communities, health professionals, local government represents, and researchers), considering the food context of the participant communities. Despite the data collection, the 24 h recall is an instrument highly recommended to collect data on food processing in consumption surveys, and it was built to collect detailed information on food processing [48]. However, caution is necessary in interpreting 24 h recall data as representative of usual intake, because they were applied once at baseline and another post-intervention.

The study has several limitations. The high prevalence of food insecurity across all communities, which was even more pronounced in those who participated in the intervention. However, sensitivity analyses, adjusting for this variable in the models, revealed no significant changes in the estimates for any of the outcomes. Additionally, there were follow-up losses within an already small sample. The intervention duration, the number of workshops, and the decision to limit workshop participation to a smaller group of individuals with potential for wider dissemination were logistical necessities but may have restricted the intervention’s reach across the entire community. Since quilombola communities present some heterogeneity, our results should be interpreted with caution. Finally, the short-term nutritional counseling intervention may be insufficient to produce changes in the eating habits of individuals inserted in a complex scenario of the food system, socioeconomic vulnerability, and high prevalence of food and nutritional insecurity, which has as a background structural racism [15]. Structural racism is a barrier to healthcare and a risk factor for illness of the Black population. A systematic review [49] found that perceived racial discrimination is associated with an unhealthy dietary pattern, characterized by higher consumption of sweets and fats and lower consumption of fruits and vegetables. The conceptual framework proposed by the authors of that systematic review to explain the relationship between racial discrimination and eating habits highlighted the interactions between social and biological mechanisms [49].

In conclusion, we found that a nutrition counseling intervention is likely to promote healthy food habits by rescuing traditional culinary preparations in quilombola communities in the South of Brazil. However, changes in the consumption of minimally processed foods were not found. Furthermore, the intervention did not mitigate the increasing trend of ultra-processed foods in quilombolas’ diet. In this sense, our findings raise the hypothesis that to obtain substantial changes in the food consumption of subgroups with high social vulnerability, intervention studies, and public policies need to act on distinct variables of the food system.
