# Supplementary material for: Effectiveness of a Nutrition Counseling Intervention on Food Consumption, According to the Degree of Processing: A Community-Based Non-Randomized Trial of Quilombola Communities in South Brazil
Source: Int J Public Health. 2024 Nov 27;69:1607549. doi: 10.3389/ijph.2024.1607549 (PMC11631577; doi:10.3389/ijph.2024.1607549)
Supplement: Supplementary file 1 [file DataSheet1.pdf]

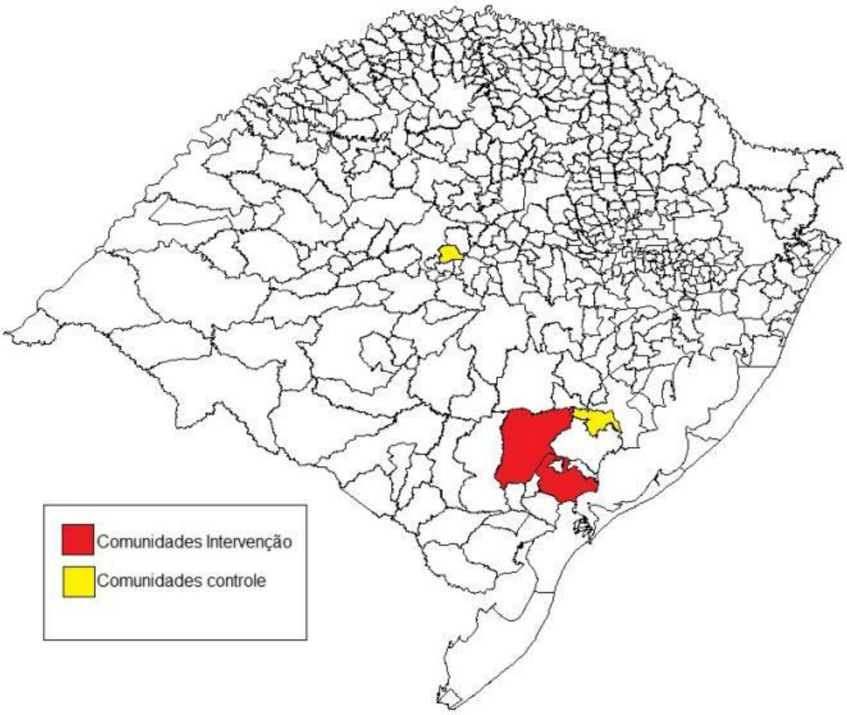

Source: IBGE cartographic base.

Supplementary Figure 1. Geographic distribution of intervention and control communities in the State of Rio Grande do Sul, Brazil, 2014-2016.

Supplementary Table 1. Themes table of the workshops held.

Themes of the workshops		Objectives
Workshop 1	Cultural and Ethnic Identity	To encourage participants to reflect on their ethnic and racial affiliation, as well as to understand the social representation that quilombolas have of themselves.
Workshop 2	Industrialized “food” and our health	To raise awareness about the relationship between excess sodium, sugar, and lipids and health, to demonstrate the amount of these substances in various processed foods, and to encourage the reduction of their consumption.
Workshop 3	Culinary Workshop: preparations with reduced salt, sugar, and fat	The objectives of this workshop were to raise awareness about the use of natural spices and to encourage the preparation of dishes with reduced fat and sugar. The following topics were covered: natural spices, their applications and benefits, and preparations with reduced salt, sugar, and fat.
Workshop 4	Reviewing Concepts: Demystifying traditional foods and preparations	To reduce the stigma surrounding some traditional and healthy foods and preparations from the past, and to give them new significance in modern preparation methods, thereby reintegrating them into everyday life.
Workshop 5	Food as Heritage	To promote the recognition of food as cultural heritage. The topics covered included traditional foods and recipes, food as memory and affection, and the preservation of traditional foods and recipes.
Workshop 6	Access to Food Security Programs	To increase participants' knowledge about programs to combat hunger, through a conversation about their objectives and conditions for access.

Source: Neutzling,2014
